# Nuclear Receptors in Health and Diseases

**DOI:** 10.3390/ijms24119153

**Published:** 2023-05-23

**Authors:** Pengfei Xu

**Affiliations:** 1 Department of Hepatobiliary and Pancreatic Surgery, Zhongnan Hospital of Wuhan University, School of Pharmaceutical Sciences, Wuhan University, Wuhan 430071, China; pengfeixu@whu.edu.cn; 2 Center for Pharmacogenetics and Department of Pharmaceutical Sciences, University of Pittsburgh, Pittsburgh, PA 15261, USA

Nuclear receptors (NRs) are a vital superfamily of transcription factors that play crucial roles in physiology and pharmacology [1]. The human genome comprises 48 NRs, 24 of which are ligand-dependent transcription factors [2]. The NRs in humans are categorized into six subgroups based on their sequence homology and structure [1]: thyroid-hormone-receptor-like, retinoid-x-receptor-like, estrogen-receptor-like, nerve-growth-factor-IB-like, steroidogenic-factor-like, and germ-cell-nuclear-factor-like, which are presented in Table 1. These NRs play significant roles in various processes, including development, reproduction, aging, and cancer [3]. They act as key mediators in maintaining everyday health by regulating metabolism and circadian rhythms. NRs can directly bind to specific promoter elements on DNA to regulate the expression of target genes, which, in turn, control metabolism, homeostasis, development, and reproduction.

Most NRs consist of six functional domains [4], including the variable N-terminal regulatory domain (A–B), retained DNA-binding domain (DBD) (C), hinge region (D), conserved ligand-binding domain (LBD) (E), and variable C-terminal domain (F), as illustrated in Figure 1A. NRs are transcription factors that are activated by ligands, small lipid-based molecules, to regulate gene expression and cellular processes. This family comprises receptors (class I) for various steroid hormones and their derivatives, including estrogen, progesterone, glucocorticoids, vitamin D, oxysterols, and bile acids, as well as receptors for retinoic acids, thyroid hormones, fatty acids, and their derivatives [5]. Some NRs are cytoplasmic in the absence of ligands and form complexes with heat shock proteins (HSP) that regulate their cellular location, protein stability, steroid-hormone-binding capacity, and transcriptional activity [6]. Upon ligand binding, these receptors undergo dimerization and translocate to the nucleus, as depicted in Figure 1B. Optionally, class II NRs are retained in the nucleus and their chromatin-modifying activities are modulated by ligand binding to form heterodimers (usually with retinoid X receptors (RXR)) [7], as depicted in Figure 1C. NRs function primarily by transcriptionally regulating the expression of related target genes through the recruitment of coactivators or corepressors when ligands bind to the receptors [8]. NRs are highly druggable targets due to their ability to bind and be activated by a large number of small molecules, with 10–20% of FDA-approved medications currently targeting NRs [9]. These medications have an estimated market value of 30 billion dollars per year. Consequently, a variety of opinions exist for repurposing these approved medications and developing new therapies targeting NRs for numerous diseases.

The interaction between NRs and other biological processes presents an exciting area of research. DNA damage repair pathways are intimately connected to steroid signaling through transcriptional processes, which has implications for drug combinations in a variety of diseases. The previously unknown roles of NRs in epigenetic regulation and their involvement in essential signaling pathways and metabolic mechanisms indicate that this superfamily has the potential not only to affect development and normal functioning, but also to contribute to disease development. This Special Issue conveys several prominent researchers in the field of nuclear receptor research from around the world to examine the emerging roles of NRs (mainly including farnesoid X receptors (FXR), peroxisome proliferator-activated receptors (PPARs), constitutive activated receptors (CAR), estrogen-related receptor γ (ERRγ), and RXR, etc.) and their impact on health maintenance and disease pathogenesis, such as cancers, metabolic disorders, growth and development, inflammation, Parkinson’s disease, and kidney disease.

FXR, also referred to as NR1H4, is a nuclear receptor that responds to bile acid and plays a role in regulating the metabolisms of bile acid, lipid, and glucose to maintain homeostasis [10]. Several studies have suggested the role of FXR in regulating genes that manipulate intestinal glucose [11,12,13]. Jiufang et al. investigated the role of gut-specific FXR in glucose absorption using a unique double-tagged glucose kinetic approach in FXR^△gut^ mice [14]. Although the FXR^△gut^ mice exhibited a reduced expression of hexokinase 1 (Hk1) in the duodenum when subjected to an obesogenic challenge, the analysis of the glucose fluxes in these mice did not indicate that gut FXR plays a role in glucose absorption. These findings suggest that the delayed glucose uptake observed in FXR-deficient mice is not due to a lack of FXR in the intestine [14]. However, intestinal FXR is involved in regulating the surface area of the small intestine. Jae Man et al. reported that the gene encoding the transient receptor potential melastatin 6 (TRPM6), which is an Mg^2+^ channel, could be a direct target of the FXR in the intestinal epithelial cells of mice. The maintenance of the basal expression of intestinal TRPM6 in the presence of GW4064, an artificial FXR agonist, is dependent on the FXR expression in the intestinal epithelial cells [15]. Additionally, they proposed a gut FXR–TRPM6 axis that could link bile acid signaling to Mg^2+^ homeostasis. Yongdong Niu’s lab found that HBx C40, a C-terminal truncated X protein and known FXR-binding protein, contributes to tumor cell proliferation and migration by modifying the distribution of the cell cycle and causing apoptosis, independent of FXR. HBx C40 enhances the growth of FXR deficiency tumors in vivo by modifying the cellular cycle distribution and disrupting the glucose metabolism to promote the development of hepatocellular carcinoma [16]. FXR is also known to play a critical role in renal water reabsorption and is believed to provide a protective effect in both acute and chronic renal diseases, including those related to diabetes. Xiaoyan Zhang’s laboratory has summarized the recent developments in understanding the physiological and pathophysiological roles of FXR in the kidney [17].

Non-steroidal anti-inflammatory drugs (NSAIDs) are considered to be cancer prevention therapeutics due to their ability to restrict cyclooxygenases (COX), which are commonly overexpressed in various types of cancer [18]. Adam et al. demonstrated that NSAIDs can induce anti-neoplastic activity by activating PPARγ, which, in turn, induces proline dehydrogenase/proline oxidase (PRODH/POX)-dependent apoptosis [19]. Therefore, targeting proline metabolism and the PRODH/POX–PPARγ axis could be a potential new approach for treating breast cancer [19]. Lsaac et al. investigated the therapeutic effects of PPARs, particularly the gamma isoform (PPARγ), in preclinical animal models of Parkinson’s disease (PD) and clinical trials for PD [20]. They suggested the possible anti-α-synucleinopathy mechanisms downstream from these NRs [20]. Understanding the neuroprotective mechanisms of PPARs, using preclinical models that mimic PD as closely as possible, can facilitate better clinical trials for disease-modifying medications in PD [20]. Fenofibrate, a widely used cholesterol-lowering agent, acts as a ligand of PPARα [21]. Katerina et al. investigated the impact of fenofibrate on the activated p38 (p-p38) levels in colorectal cancer cell lines with differentiating statuses. They found that fibrates upregulated the p-p38 in undifferentiated HT-29 cells, whereas, in other cases, the p-p38 expression was reduced. The authors also observed that PPAR ligands affected the expression of the p-p38 in the cytoplasm of HT-29 cells, whereas, in Caco2 cells, fibrates affected the p-p38 expression in both the cytoplasm and nucleus [22].

The xenobiotic receptor CAR is activated by a variety of environmental chemicals and participates in xenobiotic metabolism [23,24]. The Yonggong Zhai lab investigated the effects of maternal exposure to the CAR-specific ligand 1,4-bis [2-(3,5-dichloropyridyloxy)] benzene (TCPOBOP) on offspring health outcomes [25]. Maternal TCPOBOP exposure led to a greater inhibition of body weight in first-generation (F1) female mice compared to the control group. Additionally, maternal exposure to TCPOBOP resulted in a significant increase in the hepatic-drug-metabolizing enzyme expression in F1 generation [25]. Mechanistically, maternal TCPOBOP exposure triggered the intestinal inflammatory response and disrupted the antioxidant system in the offspring female mice, which hindered the absorption of nutrients in the intestine and posed a serious threat to the health of their offspring [25].

ERRγ, an orphan nuclear receptor, is a crucial transcription factor that plays a significant role in mitochondrial metabolism and energy homeostasis. Soon-Young and colleagues conducted cell-based experiments and demonstrated that GSK5182, a specific reverse agonist of ERRγ, can impede the ubiquitination of ERRγ, thereby stabilizing the protein [26]. The researchers also found that GSK5182’s stabilization of ERRγ is dependent on the presence of Y326, as a mutation of this amino acid to alanine (Y326A) prevented a ligand-induced stabilization of the protein. Furthermore, GSK5182 hindered the ubiquitination of ERRγ via the E3 Parkin ligase, which prevented its subsequent degradation [26].

Megumi et al. found that the RXR ligand, all-trans retinoic acid (ATRA), induced synergistic and beneficial effects on TGF-β2-treated human trabecular meshwork (HTM) cells cultured in both 2D and 3D environments. Notably, the effects varied considerably between the two cultures [27].

Valentina and colleagues provided a comprehensive summary of the role of nuclear receptors (NRs) in erythropoiesis, covering their involvement in both homeostatic and stressful conditions [28]. This knowledge is essential for regulating the production of red blood cells, particularly in disease conditions such as anemia. Additionally, this understanding is crucial for expanding erythroid cells in culture, enabling the long-term objective of cultured blood for transfusions.

The current Special Issue serves as an open-access platform for a collection of reviews and original research articles that discuss the advanced features of NRs in physiological and pathological states. These articles provide a fascinating resource on the various aspects of NRs, ranging from fundamental science to applied therapeutic approaches.

## Figures and Tables

**Figure 1:**
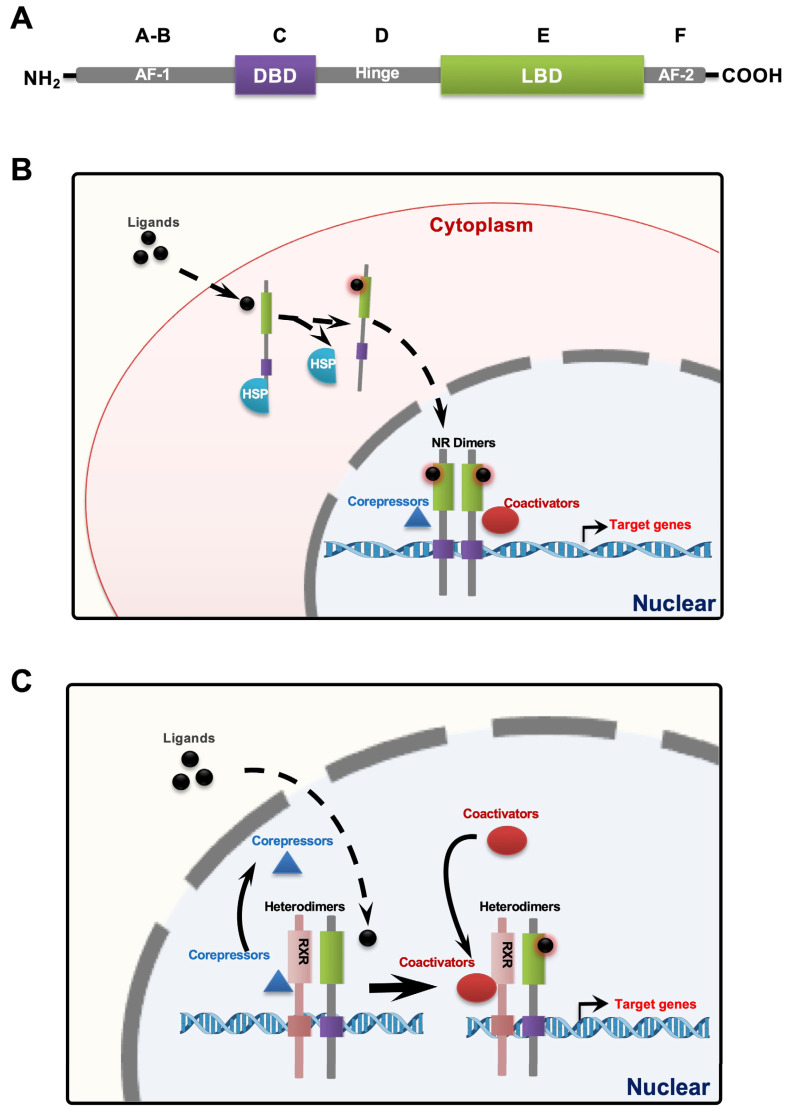
Schematic structure of NRs (nuclear receptors) and model of NR signaling. (**A**) General domain structure of NRs; (**B**) mechanism of class I NR action; and (**C**) mechanism of class II NR action.

**Table 1:** A summary of different NRs categorized according to their structure and sequence homology.

Subfamily	Number	Group	NRNC Symbol	Abbreviation	Name
**1. Thyroid Hormone Receptor-like**	1.1	Thyroid hormone receptor	NR1A1	TRα	Thyroid hormone receptor-α
			NR1A2	TRβ	Thyroid hormone receptor-β
	1.2	Retinoic acid receptor	NR1B1	RARα	Retinoic acid receptor-α
			NR1B2	RARβ	Retinoic acid receptor-β
			NR1B3	RARγ	Retinoic acid receptor-γ
	1.3	Peroxisome proliferator-activated receptor	NR1C1	PPARα	Peroxisome proliferator-activated receptor-α
			NR1C2	PPAR-β/δ	Peroxisome proliferator-activated receptor-β/δ
			NR1C3	PPARγ	Peroxisome proliferator-activated receptor-γ
	1.4	Rev-ErbA	NR1D1	Rev-ErbAα	Rev-ErbAα
			NR1D2	Rev-ErbAβ	Rev-ErbAβ
	1.5	RAR-related orphan receptor	NR1F1	RORα	RAR-related orphan receptor-α
			NR1F2	RORβ	RAR-related orphan receptor-β
			NR1F3	RORγ	RAR-related orphan receptor-γ
	1.6	Liver X receptor-like	NR1H2	LXRβ	Liver X receptor-β
			NR1H3	LXRα	Liver X receptor-α
			NR1H4	FXR	Farnesoid X receptor
	1.7	Vitamin D receptor-like	NR1I1	VDR	Vitamin D receptor
			NR1I2	PXR	Pregnane X receptor
			NR1I3	CAR	Constitutive androstane receptor
**2. Retinoid X Receptor-like**	2.1	Hepatocyte nuclear factor-4	NR2A1	HNF4α	Hepatocyte nuclear factor-4-α
			NR2A2	HNF4γ	Hepatocyte nuclear factor-4-γ
	2.2	Retinoid X receptor	NR2B1	RXRα	Retinoid X receptor-α
			NR2B2	RXRβ	Retinoid X receptor-β
			NR2B3	RXRγ	Retinoid X receptor-γ
	2.3	Testicular receptor	NR2C1	TR2	Testicular receptor 2
			NR2C2	TR4	Testicular receptor 4
	2.4	TLX/PNR	NR2E1	TLX	Homologue of the Drosophila tailless gene
			NR2E3	PNR	Photoreceptor cell-specific nuclear receptor
	2.5	COUP/EAR	NR2F6	EAR-2	V-erbA-related
**3. Estrogen Receptor-like**	3.1	Estrogen receptor	NR3A1	ERα	Estrogen receptor-α
			NR3A2	ERβ	Estrogen receptor-β
	3.2	Estrogen related receptor	NR3B1	ERRα	Estrogen-related receptor-α
			NR3B2	ERRβ	Estrogen-related receptor-β
			NR3B3	ERRγ	Estrogen-related receptor-γ
	3.3	3-Ketosteroid receptors	NR3C1	GR	Glucocorticoid receptor
			NR3C2	MR	Mineralocorticoid receptor
			NR3C3	PR	Progesterone receptor
			NR3C4	AR	Androgen receptor
**4. Nerve Growth Factor IB-like**	4.1	NGFIB/NURR1/NOR1	NR4A1	NGFIB	Nerve Growth factor IB
			NR4A2	NURR1	Nuclear receptor related 1
			NR4A3	NOR1	Neuron-derived orphan receptor 1
**5. Steroidogenic Factor-like**	5.1	SF1/LRH1	NR5A1	SF1	Steroidogenic factor 1
			NR5A2	LRH-1	Liver receptor homolog-1
**6. Germ Cell Nuclear Factor-like**	6.1	GCNF	NR6A1	GCNF	Germ cell nuclear factor

## References

[1] Auwerx J., Baulieu E., Beato M., Becker-Andre M., Burbach P.H., Camerino G., Chambon P., Cooney A., Dejean A., Dreyer C. (1999). A unified nomenclature system for the nuclear receptor superfamily. Cell.

[2] Mangelsdorf D.J., Thummel C., Beato M., Herrlich P., Schütz G., Umesono K., Blumberg B., Kastner P., Mark M., Chambon P. (1995). The nuclear receptor superfamily: The second decade. Cell.

[3] Gronemeyer H., Gustafsson J.Å., Laudet V. (2004). Principles for modulation of the nuclear receptor superfamily. Nat. Rev. Drug Discov..

[4] Xu P., Zhai Y., Wang J. (2018). The Role of PPAR and Its Cross-Talk with CAR and LXR in Obesity and Atherosclerosis. Int. J. Mol. Sci..

[5] Bain D.L. (2007). Nuclear receptor structure: Implications for function. Annu. Rev. Physiol..

[6] Echeverria P.C., Picard D. (2010). Molecular chaperones, essential partners of steroid hormone receptors for activity and mobility. Biochim. Biophys. Acta.

[7] Lazar M.A. (2017). Maturing of the nuclear receptor family. J. Clin. Investig..

[8] Hong F., Pan S., Guo Y., Xu P., Zhai Y. (2019). PPARs as Nuclear Receptors for Nutrient and Energy Metabolism. Molecules.

[9] Kinch M.S., Hoyer D., Patridge E., Plummer M. (2015). Target selection for FDA-approved medicines. Drug Discov. Today.

[10] Sun L., Cai J., Gonzalez F.J. (2021). The role of farnesoid X receptor in metabolic diseases, and gastrointestinal and liver cancer. Nat. Rev. Gastroenterol. Hepatol..

[11] Zheng X., Chen T., Jiang R., Zhao A., Wu Q., Kuang J., Sun D., Ren Z., Li M., Zhao M. (2021). Hyocholic acid species improve glucose homeostasis through a distinct TGR5 and FXR signaling mechanism. Cell Metab..

[12] Sun L., Xie C., Wang G., Wu Y., Wu Q., Wang X., Liu J., Deng Y., Xia J., Chen B. (2018). Gut microbiota and intestinal FXR mediate the clinical benefits of metformin. Nat. Med..

[13] Chávez-Talavera O., Tailleux A., Lefebvre P., Staels B. (2017). Bile Acid Control of Metabolism and Inflammation in Obesity, Type 2 Diabetes, Dyslipidemia, and Nonalcoholic Fatty Liver Disease. Gastroenterology.

[14] Yang J., van Dijk T.H., Koehorst M., Havinga R., de Boer J.F., Kuipers F., van Zutphen T. (2023). Intestinal Farnesoid X Receptor Modulates Duodenal Surface Area but Does Not Control Glucose Absorption in Mice. Int. J. Mol. Sci..

[15] Kim E.Y., Lee J.M. (2022). Transcriptional Control of Trpm6 by the Nuclear Receptor FXR. Int. J. Mol. Sci..

[16] Wu X., Ni Z., Song T., Lv W., Chen Y., Huang D., Xie Y., Huang W., Niu Y. (2023). C-Terminal Truncated HBx Facilitates Oncogenesis by Modulating Cell Cycle and Glucose Metabolism in FXR-Deficient Hepatocellular Carcinoma. Int. J. Mol. Sci..

[17] Guo Y., Xie G., Zhang X. (2023). Role of FXR in Renal Physiology and Kidney Diseases. Int. J. Mol. Sci..

[18] Zappavigna S., Cossu A.M., Grimaldi A., Bocchetti M., Ferraro G.A., Nicoletti G.F., Filosa R., Caraglia M. (2020). Anti-Inflammatory Drugs as Anticancer Agents. Int. J. Mol. Sci..

[19] Kazberuk A., Chalecka M., Palka J., Surazynski A. (2022). Nonsteroidal Anti-Inflammatory Drugs as PPARgamma Agonists Can Induce PRODH/POX-Dependent Apoptosis in Breast Cancer Cells: New Alternative Pathway in NSAID-Induced Apoptosis. Int. J. Mol. Sci..

[20] Pérez-Segura I., Santiago-Balmaseda A., Rodríguez-Hernández L.D., Morales-Martínez A., Martínez-Becerril H.A., Martínez-Gómez P.A., Delgado-Minjares K.M., Salinas-Lara C., Martínez-Dávila I.A., Guerra-Crespo M. (2023). PPARs and Their Neuroprotective Effects in Parkinson’s Disease: A Novel Therapeutic Approach in alpha-Synucleinopathy?. Int. J. Mol. Sci..

[21] Hong F., Xu P., Zhai Y. (2018). The Opportunities and Challenges of Peroxisome Proliferator-Activated Receptors Ligands in Clinical Drug Discovery and Development. Int. J. Mol. Sci..

[22] Cizkova K., Tauber Z. (2023). Fibrates Affect Levels of Phosphorylated p38 in Intestinal Cells in a Differentiation-Dependent Manner. Int. J. Mol. Sci..

[23] Xu P., Hong F., Wang J., Dai S., Wang J., Zhai Y. (2018). The CAR agonist TCPOBOP inhibits lipogenesis and promotes fibrosis in the mammary gland of adolescent female mice. Toxicol. Lett..

[24] Pan S. (2023). Environmental chemical TCPOBOP disrupts milk lipid homeostasis during pregnancy and lactation. Ecotoxicol. Environ. Saf..

[25] Pan S., Guo Y., Yu W., Zhang J., Qiao X., Li L., Xu P., Zhai Y. (2023). Constitutive Androstane Receptor Agonist, TCPOBOP: Maternal Exposure Impairs the Growth and Development of Female Offspring in Mice. Int. J. Mol. Sci..

[26] Na S.Y., Kim K.S., Jung Y.S., Kim D.K., Kim J., Cho S.J., Lee I.K., Chung J., Kim J.S., Choi H.S. (2022). An Inverse Agonist GSK5182 Increases Protein Stability of the Orphan Nuclear Receptor ERRgamma via Inhibition of Ubiquitination. Int. J. Mol. Sci..

[27] Watanabe M., Sato T., Tsugeno Y., Higashide M., Furuhashi M., Umetsu A., Suzuki S., Ida Y., Hikage F., Ohguro H. (2022). All-trans Retinoic Acids Synergistically and Beneficially Affect In Vitro Glaucomatous Trabecular Meshwork (TM) Models Using 2D and 3D Cell Cultures of Human TM Cells. Int. J. Mol. Sci..

[28] Pastori V., Pozzi S., Labedz A., Ahmed S., Ronchi A.E. (2022). Role of Nuclear Receptors in Controlling Erythropoiesis. Int. J. Mol. Sci..

